# Pharmacological Overview of Bioactive Natural Products from *Gynura procumbens* (Lour.) Merr

**DOI:** 10.3390/plants14172714

**Published:** 2025-09-01

**Authors:** Ponkti Addrita Bose, Md Mehadi Hasan Sohag, Muhammad Fazle Rabbee, Tareque Muzahid Zamee, Jab-un-nisha Kona, Bonhi Elora, Randa Mohammed Zaki, Kamrul Islam, Kwang-Hyun Baek

**Affiliations:** 1 Department of Genetic Engineering and Biotechnology, Jagannath University, Dhaka 1100, Bangladesh; addritaponkti@gmail.com (P.A.B.); mmhasansohag@geb.jnu.ac.bd (M.M.H.S.); 2 Biotechnology Research Initiative for Sustainable Development, Dhaka 1219, Bangladesh; 3 Department of Biotechnology, Yeungnam University, Gyeongsan 38541, Republic of Korea; rabbi.biotech@gmail.com; 4 Department of Microbiology, University of Dhaka, Dhaka 1000, Bangladesh; tmzamee@gmail.com; 5 Department of Genetic Engineering and Biotechnology, Shahjalal University of Science and Technology, Sylhet 3114, Bangladesh; jab.un.nisha.kona@gmail.com; 6 Anandaniketan, Sylhet 3100, Bangladesh; bonhielora@gmail.com; 7 Department of Pharmaceutics, College of Pharmacy, Prince Sattam Bin Abdulaziz University, P.O. Box 173, Al Kharj 11942, Saudi Arabia; r.abdelrahman@psau.edu.sa; 8 Department of Pharmaceutics and Industrial Pharmacy, Faculty of Pharmacy, Beni-Suef University, Beni-Suef 62514, Egypt

**Keywords:** medicinal plant, phytochemicals, flavonoids, natural remidies, *Gynura procumbens*

## Abstract

*Gynura procumbens* is a commonly adopted medicinal plant native to the tropical regions of East and Southeast Asia and is well recognized for its significant therapeutic potential. Traditionally used in herbal medicine, it has been employed to manage various conditions, including hypertension, diabetes, renal disorders, constipation, and inflammation. Its leaves are particularly rich in flavonoids—such as astragalin, kaempferol, quercetin, myricetin, and rutin—which are associated with anti-glycaemic, anticancer, antihypertensive, antimicrobial, and antioxidant activities. These bioactive constituents form the basis of the broad pharmacological profile of the plant. Emerging studies also suggest a potential role for *G. procumbens* in the management of infertility, further broadening its therapeutic scope. This review provides a concise overview of its phytochemical constituents, taxonomic classification, and current pharmacological evidence, highlighting its potential as a valuable candidate for novel drug development.

## 1. Introduction

In recent years, changes in lifestyle have led to a rise in chronic diseases, such as hypertension, diabetes, cardiovascular diseases, and cancer. Treating these conditions is often expensive and may cause significant adverse side effects [1]. Therefore, allopathic remedies are frequently used, especially in Asia, where these diseases are highly prevalent [2]. However, many individuals increasingly prefer therapeutic regimens that incorporate bioactive compounds derived from natural sources. Medicinal herbs have been used for thousands of years to prevent and treat a wide range of diseases, and approximately 80% of the global population still relies on them today [3]. Through centuries of empirical use, traditional herbal therapies have become an integral part of human healthcare practices. Various parts of medicinal plants—such as leaves, seeds, and fruits—have shown potential in promoting health and managing numerous diseases [4]. The global market for herbal supplements is projected to reach USD 160 billion by 2030, with an estimated compound annual growth rate of 8.5% [5].

**Figure 1:**
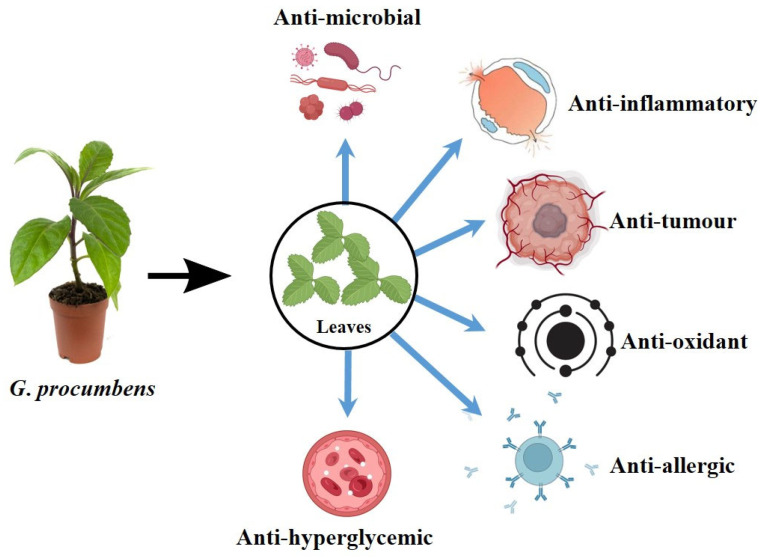
Pharmacological activities of *G. procumbens* leaf extracts.

*Gynura procumbens*, commonly known as longevity Spinach, is a fast-growing herbaceous plant valued for its rich medicinal properties and health-promoting compounds. Despite its wide range of reported pharmacological benefits, it remains underutilized. *G*. *procumbens*, indigenous to Southeast Asia, has been traditionally utilized for centuries as a functional food and herbal remedy. The leaves, in particular, are used in various culinary forms: in Thailand, they are cooked as vegetables, whereas in Malaysia, they are often eaten raw in salads, sometimes served with rice [6,7]. Historically, *G. procumbens* has been used to treat a broad range of health conditions, including fever, migraines, skin rashes, and infections accompanied by inflammation, alongside non-communicable diseases, such as hypertension, kidney disorders, high cholesterol, and cancer [8]. This plant exhibits significant therapeutic properties, including antimicrobial, antioxidant, anti-inflammatory, antihyperglycemic, antiallergic, and antitumorigenic activities (Figure 1 and Table 1). These effects are attributed to various bioactive compounds found in *G. procumbens*, such as flavonoids, terpenoids, and saponins [9]. Among these, flavonoids and other phenolic compounds are believed to play a central role in the medicinal efficacy of the plant [10,11].

The plant is believed to have originated in the African subcontinent, but it is now widely distributed across several Asian countries, including Thailand, China, Malaysia, Indonesia, Myanmar, and Vietnam [12]. *G. procumbens* propagates easily from stem cuttings when cultivated in moist, well-drained, and fertile soil. It grows best in partially shaded environments but can also adapt to full sunlight, provided that the soil around the roots remains consistently moist. During the early stages of planting, *G. procumbens* should be kept strictly in a partially shaded environment to prevent leaf scorching and stunted growth. *G. procumbens* grows to a height of 1–3 m. It has fleshy stems and leaves that are either ovate-elliptic or lanceolate in shape. The plant is known by various vernacular names depending on the region. For example, it is called Paetumpung in Thailand, Sambung Nyawa in Malaysia and Indonesia, Akar Sebiak among Chinese communities, and Longevity spinach in the United States.

Owing to the growing global demand for this plant among health-conscious individuals, several agricultural studies have been conducted to evaluate its economic potential, particularly in countries such as Indonesia [13] and Malaysia [14]. Because the growth requirements of longevity spinach are similar to those of other common tropical species, it is considered cost-effective to cultivate it in tropical regions with moist and fertile soil.

**Table 1:** Pharmacological activities of *G. procumbens* and their associated mechanisms of action.

SL No.	Activity	Mechanisms and Effects	References
1	Antioxidant	Scavenges free radicals and upregulates antioxidant enzymes (SOD, CAT, and GPx)	[6,7]
2	Anti-inflammatory	Inhibits COX-2, NF-κB, TNF-α, and IL-6 pathways	[15]
3	Antidiabetic	Enhances insulin sensitivity, lowers blood glucose, and inhibits α-glucosidase	[16]
4	Antihypertensive	Vasodilatory effect and improves endothelial function	[17]
5	Anticancer	Induces apoptosis, inhibits proliferation, angiogenesis, and metastasis	[18,19]
6	Hepatoprotective	Protects liver tissue from oxidative and chemical-induced injury	[20]
7	Antimicrobial	Active against bacteria and fungi; potentiates antibiotics	[14]
8	Hypolipidemic	Lowers total cholesterol and triglycerides; increases HDL	[21,22]
9	Wound healing	Promotes tissue regeneration and reduces inflammation	[23]

Abbreviations: SOD, superoxide dismutase; CAT, catalase; GPx, glutathione peroxidase; COX-2, cyclooxygenase-2; NF-κB, nuclear factor kappa-light chain enhancer of activated; TNF-α, Tumor necrosis factor-α; IL-6, interleukin-6; and HDL, high-density lipoprotein.

## 2. Flavonoids Synthesized by *G. procumbens*

Secondary metabolites and other bioactive substances found in medicinal plants possess significant therapeutic potential and are collectively known as phytoactive compounds. These naturally occurring compounds have played a crucial role in drug discovery and development. Several plant-derived compounds have been successfully isolated and clinically applied to treat numerous human diseases. For example, Artemisinin from *Artemisia annua* (sweet wormwood), morphine from *Papaver somniferum* (opium poppy), and paclitaxel from *Taxus brevifolia* (western yew) [24]. Phytochemical investigations have revealed that the leaves of *G. procumbens* are the primary reservoirs of its bioactive compounds, which contribute significantly to its pharmacological activities (Table 2).

**Figure 2:**
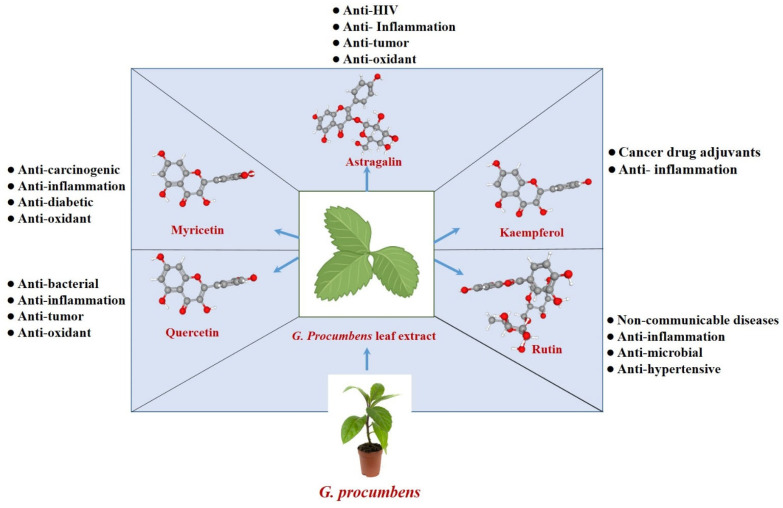
Major bioactive flavonoids in *G. procumbens* leaf extract and their associated pharmacological properties. The three-dimensional structures of Astragalin, Kaempferol, Myricetin, Quercetin, and Rutin were obtained from the PubChem database.

Among these, flavonoids and flavonoid-derived compounds are particularly abundant. Traditionally, the leaves of *G. procumbens* are used more frequently than its roots, even though several studies have shown that roots contain higher levels of phenolic compounds, flavonoids, and ascorbic acid [27]. *G. procumbens* leaf, stem, and root callus extracts exhibited total flavonoid content (TFC) of 75.258, 43.351, and 21.961 μg/g FW, respectively. In contrast, *G. procumbens* root extract shows the highest TFC with 543.529 μg/g FW [27]. However, it is important to note that this result, obtained through a colorimetric assay, can vary depending on the type and polarity of the solvent used, the extraction method, and the conditions. Therefore, further research is needed to compare different extraction methods and optimize the extraction process for *G. procumbens*. The flowers of *G. procumbens* contain β-sitosterol, aurantiamide acetate, neochlorogenic acid, chlorogenic acid, cryptochlorogenic acid, and isochlorogenic acids B, A, and C, which are also potent antioxidants [27]. A recent study also reported phytol, lupeol, stigmasterol, and friedelanol acetate extracted from *G. procumbens* for the first time [12]. Besides these, hydroxybenzoic acids, hydroxycinnamic acid, terpenoids, saponins, and flavonoids, such as astragalin, myricetin, kaempferol, rutin, and quercetin, are commonly found in *G. procumbens* leaves [6,26]. However, most of the bioactive compounds in *G. procumbens* are concentrated in its leaves, including carbohydrates, saponins, tannins, and flavonoids. Among these, flavonoids and flavonoid-derived compounds are particularly abundant. Previous studies have identified several key flavonoids in *G. procumbens* extracts and related plant species, such as kaempferol, rutin, astragalin, myricetin, and quercetin [6]. These flavonoids are solely responsible for regulating metabolic switching and vital signaling that induce the pharmacological activities (Figure 2 and Table 3).

### 2.1. Astragalin (Kaempferol-3-O-β-D-glucopyranoside)

Astragalin, a flavonoid compound also known as Kaempferol-3-O-β-D-glucopyranoside -3O is primarily isolated from *Phytolacca americana* and is also found in extracts of *Pheqopteris connectilis* fern. Chemically, astragalin is the 3-O-*β*-D-glucoside derivative of kaempferol. It exhibits a broad range of pharmacological activities, such as antioxidant, antitumor, anti-inflammatory, and anti-HIV properties [57]. Additionally, in experimental studies using mice, astragalin has demonstrated neuroprotective effects against Alzheimer’s disease. Alzheimer’s disease is one of the predominant neurodegenerative disorders, accounting for 50–70% of dementia cases globally [58].

Astragalin has been reported to exhibit therapeutic effects against various diseases, including diabetes mellitus, neuropathy, reproductive system disease, cancer, respiratory disease, mastitis, ulcerative colitis, obesity, and ischemia [59]. It has a molecular weight of approximately 448.4 g/mol, as calculated using PubChem version 2.2 (release 14 October 2021), and is found in several edible plants, including green tea seeds, *Morus alba* L., *Cuscuta chinensis* [59], and *Gynura Procumbens* [60]. To date, astragalin shows various pharmacological activities, such as anti-inflammatory, antioxidant, antioxidative, antibacterial, analgesic, and procoagulant effects [57]. Astragalin modulates the Toll-like receptor-4/Nuclear Factor kappa-light-chain-enhancer of activated B cells (TLR4/NF-κB) signaling pathway, a key immune cascade involved in the recognition of microbial infections, suppression of inflammation, and reduction of oxidative stress [61]. Astragalin also demonstrates neuroprotective effects by regulating multiple signaling pathways, including HO-1/MAPK, P13K/Akt, SIRT1, and Notch/HES-1-NF-κB [58]. Furthermore, it mediates the BMP pathway, contributing to the improvement of osteoarthritis and osteoporosis [59]. In addition, astragalin alleviates ulcerative colitis via the NF-κB pathway, which plays a central role in immune response regulation [62].

Astragalin exhibits anticancer activity against cancers of the skin, breast, liver, lung, kidney, and digestive system. It promotes programmed cell death (apoptosis) and attenuates tumor cell proliferation through multiple signalling pathways, including NF-κB, PI3K/AKT, MAPK, and JAK/STAT [59]. This flavonoid compound also downregulates the gastric epithelium glycoprotein Mucin 1 (MUC1) and other tumor-associated antigens [63]. Astragalin shows cytotoxic effects against gastric cancer cells in xenograft mouse models without inducing observable toxicity [59]. Furthermore, it prevents colon cancers by inactivating the NF-κB pathway [64]. Astragalin induces apoptosis by upregulating caspase-3, caspase-6, caspase-7, caspase-8, caspase-9, P53, and Bax, while downregulating cleaved caspase-3 and Bcl-2. Astragalin suppresses the expression of p-NF-κB, NF-κBP65, p-IκBα, TNF-α, and IL-6 [64]. Collectively, these findings suggest that astragalin plays a pivotal role in gastric carcinoma by modulating multiple associated signaling pathways. Additionally, by targeting the p38 MAPK pathways, astragalin suppresses skin carcinoma progression [59].

Astragalin regulates the estradiol metabolic pathway and suppresses the expression of angiogenesis-related proteins, thereby reducing breast cancer cell metastasis [59]. By modulating the MAPK and NF-kB signaling pathways, astragalin also inhibits the proliferation of lung cancer cells [59]. In murine studies, astragalin treatment reduced inflammation by lowering cytokine levels, regulating HO-1/MAPK and P13K/Akt signalling pathways, leading to improvements in long-term neurological function and ameliorating OVX/CUMS-induced perimenopausal depression-like behaviours via restoring IL-4R/JAK1/STAT6 pathway and inhibiting microglia-mediated neuroinflammation [65,66]. Furthermore, studies showed that astragalin also significantly inhibited the proliferation and diffusion of HCT116 cells by induced apoptosis (by modulation of Bax, Bcl-2, P53, caspase-3, caspase 6, caspase 7, caspase 8, caspase 9 protein express) and cell cycle arrest (by modulation of Cyclin D1, Cyclin E, P21, P27, CDK2, CDK4 protein express) in five human colon cancer cell lines (HCT116, LoVo, SW620, SW480, Caco2); therefore, making it a potential plant-derived antitumor drug for colon cancer [64]. Additionally, astragalin downregulates key regulators of lipid metabolism—PPAR-γ, C/EBP-α, FAS—making it effective in controlling adipogenesis, obesity, and diabetes mellitus [16,67]. In murine studies, astragalin treatment reduced inflammation by lowering cytokine levels, leading to improvements in long-term neurological function [65,66]. Furthermore, astragalin promotes rapid wound healing [23].

### 2.2. Kaempferol

Kaempferol, a naturally occurring flavonoid, is commonly found in various plants and plant-based foods. This compound is present in both dicotyledonous and monocotyledonous angiosperms, as well as in pteridophytes. With a molecular weight of 286.2 g/mol, kaempferol and its glycosides are widely distributed due to the abundance of enzymes involved in their biosynthesis in plant tissues [68]. Common edible sources include onion, chives, mustard greens, Brussels sprouts, broccoli, and kale. Consequently, *G. procumbens* contains kaempferol, similar to other medicinal plants such as Ashitaba, green chili, onion leaves, carrot, white radish, and black tea [69,70].

Kaempferol demonstrates efficacy for treating inflammatory diseases [71] and serves as an effective adjuvant in therapies for various cancers, including breast, colon, prostate, pancreatic, skin, brain, uterine, ovarian, thyroid, and bone cancers [72]. This compound is identified as a promising drug candidate in cancer treatment. In colon carcinoma, kaempferol may induce cellular differentiation [73], inhibit the progression of hormone-related cancers, such as prostate and breast cancer [74,75], and downregulate hypoxia-inducible factor-1, a key regulator of oxygen homeostasis in ovarian cancer cells [19]. Additionally, it exhibits anti-leukemic activity by inhibiting proteasome function [76]. Several studies show that dietary oral administration of kaempferol produces anti-inflammatory effects within a few days [71].

### 2.3. Myricetin

Myricetin (IUPAC name: 3,3′,4′,5,5′,7-hexahydroxyflavone), a naturally occurring flavonol widely distributed among plant species, particularly within the families *Primulaceae*, *Pinaceae*, *Myricaceae*, *Polygonaceae*, and *Anacardiaceae* [77]. Similar to various fruits and herbs, *G. procumbens* contains myricetin [26]. Myricetin exists both as a free aglycone and in glycosylated forms (e.g., myricetin-3-galactoside and myricetin3-α-l-arabinopyranoside) [77]. Owing to consistent findings from in vitro studies demonstrating its iron-chelating, antioxidant, and free radical-scavenging properties, myricetin is currently considered a promising therapeutic agent for treating and preventing various disorders, such as diabetic osteoporosis, skin and hepatocellular carcinomas, and alcoholic liver disease [18,78,79].

To date, myricetin shows potential anticancer effects against various malignancies, including ovarian cancer, choriocarcinoma, human papillary thyroid cancer, hepatocellular carcinoma, colon cancer, human synovial sarcoma, cervical cancer, and human placental choriocarcinoma, through diverse molecular mechanisms [80]. Owing to its structural similarity to adenosine triphosphate (ATP), myricetin can mimic ATP and subsequently target ATPases and protein kinases to demonstrate anticancer effects [81]. Several studies show that myricetin inhibits protein kinases via both competitive and non-competitive mechanisms [81]. In pancreatic and colon cancers, myricetin exhibits anticancer activity by inhibiting PI3K [82,83]. This inhibition may lead to tumor regression and decreased metastatic spread in vivo [82].

Myricetin exhibits a significant antiproliferative effect by inducing cytotoxicity and promoting DNA condensation in human papillary thyroid cancer cells [84]. In choriocarcinoma cells, myricetin reduces free radicals, inhibits lipid peroxidation, prevents glutathione depletion, and disrupts mitochondrial membrane potential [85]. Additionally, myricetin reduces cell proliferation by approximately two-thirds in human anaplastic thyroid cancer cells [86]. Myricetin exhibits anti-leukemic activity by interfering with the purine nucleotide biosynthesis pathway. It blocks the conversion of inosine monophosphate to xanthosine monophosphate by inhibiting the catalytic activity of human inosine 5′-monophosphate dehydrogenase [87]. A study suggests that myricetin may act as a potent chemosensitizer, enhancing the sensitivity of cancer cells to chemotherapy and thereby improving overall cancer management [88]. Furthermore, myricetin increases the radiosensitivity of lung cancer cells (A549 and H1299), leading to enhanced apoptosis following radiotherapy [89]. Compared to the other flavonoids previously mentioned, myricetin consistently exhibits anticancer activity across all reported studies.

Myricetin demonstrates antimicrobial activity against several pathogenic microorganisms, primarily bacteria and viruses. Studies show that myricetin exhibits toxicity toward HIV, methicillin-resistant *Staphylococcus aureus*, and other clinically significant microbes [90,91]. In *Escherichia coli*, myricetin inhibits DnaB helicase, a key enzyme involved in DNA replication and elongation [92]. Additionally, it stimulates epithelial chloride secretion, potentially contributing to the prevention of viral and bacterial infections [93].

Myricetin regulates glycemia, with supporting evidence from both in vitro and animal models. A study shows that myricetin can attenuate hyperglycaemia by enhancing glucose uptake in muscle and liver tissues, significantly stimulating hepatic glycogen synthase I activity and thereby promoting glycogen synthesis in hepatocytes of diabetic rats [94]. Furthermore, myricetin may modulate the glucose transport system by downregulating apical glucose transporter 2 expression in *Xenopus laevis* oocytes. This mechanism represents a key glycemic regulatory pathway, potentially inhibiting glucose absorption and increasing insulin sensitivity [95]. In another study based on diet-induced obesity in a rat model, myricetin was found to upregulate peroxisome proliferator-activated receptor-alpha and downregulate sterol regulatory element-binding proteins, leading to reduced body weight and improved lipid profiles [96]. Myricetin exhibits antioxidant activity and confers protection against inflammation [77]. Lastly, it inhibits the formation of amyloid-beta fibrils and neurofibrillary tangles, two key pathological features associated with the progression of AD, highlighting its potential as a therapeutic agent for AD [97,98].

### 2.4. Quercetin

Quercetin and its derivatives, belonging to the flavonoid class of naturally occurring phytochemicals, demonstrate significant therapeutic potential in managing various pathological conditions, including rheumatic disorders, cardiovascular diseases, AD, and several types of cancer [99,100]. These compounds exhibit antioxidant, anti-inflammatory, and antibacterial properties [101,102,103]. In nature, quercetin occurs in its free form (aglycone) or as glycoside conjugates bound to sugar molecules. The most common naturally occurring form is quercetin-3-O-glucoside; however, other glycosylated forms exist [101]. Quercetin is typically abundant in apples and onions, and present in lower concentrations in pepper, fennel, coriander, and tea. The compound is currently incorporated into various nutraceutical products [104].

Along with other flavonoids, *G. procumbens* contains quercetin, which contributes to its medicinal properties by reducing the proliferation rate and inhibiting the migration of aortic smooth muscle cells [105]. This activity stimulates apoptosis and confers a significant anti-carcinogenic effect. Several studies show that quercetin decreases tumor cell adhesion properties [106,107], highlighting its potential role in cancer management. Furthermore, quercetin inactivates oncogenes involved in cancer development, thereby inhibiting tumor growth [108]. Quercetin is an effective conjugate in several anticancer drug formulations [109]. Quercetin exhibits therapeutic effects against breast, gastric, prostate, and ovarian cancers [110,111,112]. Additionally, it can induce non-apoptotic necroptosis, making it a potential candidate for treating apoptosis-resistant cancers [113]. Quercetin offers protective effects against oxidative stress in neuronal cell lines, supporting its therapeutic potential in Parkinson’s disease [114].

Quercetin reduces the expression of adhesion molecules and other inflammatory mediators [115]. It acts as a vasodilator by relaxing the vascular smooth muscles, thereby improving blood flow. Additionally, it inhibits platelet aggregation, reducing the risk of several cardiovascular diseases [116]. Quercetin regulates the activity of cholesterol 7α-hydroxylase, an enzyme responsible for the conversion of cholesterol to bile acids [21]. A study reports that quercetin administration for approximately 3.5 months reduces oxidative stress in rats, subsequently attenuating atherosclerosis progression [117]. Additionally, it demonstrates efficacy in lowering blood triglyceride levels in rats [118]. Quercetin, as a chemical compound, contains multiple monoglycoside forms [119]. Furthermore, it forms complexes with metal ions, exhibiting independent therapeutic properties [120]. Quercetin acts as an iron chelator, protecting cells from oxidative damage [121]. This compound scavenges free radicals and prevents cell death associated with DNA and membrane damage [122]. Quercetin exhibits a significant antihypertensive effect, potentially through the inhibition of histamine and other mediators involved in allergenic responses [123]. Furthermore, quercetin is a well-established phosphodiesterase-4 inhibitor and may offer therapeutic benefits for patients with asthma [124].

### 2.5. Rutin

The primary dietary sources of the flavonoid glycoside rutin include buckwheat, black tea, apples, and black tea leaves. Studies show that administering rutin to diabetic mice reduces blood glucose levels and alleviates colon lesions by regulating the gut microbiota. Additionally, it improves blood levels of triglycerides, low-density lipoprotein, high-density lipoprotein, and total cholesterol in diabetic mice [22]. Rutin, known as vitamin P or rutoside, is commonly used as a nutraceutical agent and is regarded for its potent antioxidant properties [125]. Beyond these properties, rutin exhibits chemopreventive and chemosensitizing effects, potentially preventing cancer progression or recurrence and enhancing the efficacy of chemotherapy [126]. Supplementation with rutin is associated with improved health outcomes in individuals with type 2 diabetes mellitus (T2DM) and its related complications [127]. Rutin is abundantly found in plants belonging to different families, including *Rutaceae*, *Myrtaceae*, and *Fabaceae* [128]. The target plant in this study, *G. procumbens*, is one of such species [7]. Free radicals, which are byproducts of various biochemical reactions in the human body, exhibit harmful effects on health by disrupting metabolic processes and contributing to conditions such as carcinogenesis [129]. Similar to other flavonoids, rutin effectively scavenges free radicals and chelates metal ions, thereby exhibiting strong antioxidant properties. These antioxidant effects, primarily through free radical scavenging, underlie its antimicrobial, anti-inflammatory, anti-asthmatic, and antitumor activities [128]. Studies show that rutin inhibits lipid peroxidation and promotes physiological antioxidant defense mechanisms in diabetic rats [25].

Plant extract containing rutin demonstrates bactericidal properties against Gram-negative bacteria such as *Pseudomonas aeruginosa*, *Klebsiella pneumoniae*, and *Salmonella enteritidis*, similar to other flavonoids such as quercetin, kaempferol, and myricetin [130]. Rutin improves the microbiological quality of chicken soup by inhibiting the growth of the foodborne, drug-resistant pathogens, such as *S. aureus* and *E*. *coli* [131]. Additionally, rutin exhibits antiviral activity against several virus-associated diseases, including herpes, SARS-CoV-2, and respiratory infections [132,133]. This antiviral effect is associated with their mechanisms of inhibiting viral polymerase activity [134].

In chronic T lymphocyte-dependent colitis, rutin decreases intestinal inflammation by interacting with mucosal and lymph node T cells and by releasing quercetin. Studies suggest that rutin may be effective for treating inflammatory bowel disease (IBD) when administered at the recommended dosage [15]. This finding indicates that rutin could play a significant role in the management of IBD. Furthermore, rutin modulates several signaling pathways, thereby reducing reactive oxygen species (ROS)-associated oxidative stress and inflammation in rats [135], an effect associated with its ability to minimize brain damage and improve neurological functions [136]. Furthermore, rutin demonstrates anti-carcinogenic properties in different studies. It induces cell cycle arrest and promotes apoptosis, facilitating the elimination of abnormal cells [137]. It inhibits the proliferation of HepG2 cells, supporting its potential role in liver cancer management [138]. A study reports that rutin, as a flavonoid, enhances iodine uptake in rats, supporting its potential use as an adjuvant in radioiodine therapy [139]. Iodine uptake is essential for the biosynthesis of thyroid hormones and serves as a key indicator of different thyroid disorders. Additionally, rutin shows promise as a candidate for developing novel therapeutic prodrugs for neuroblastoma due to its ability to induce apoptosis [140].

Rutin is widely recognized for its potential in managing diabetes due to its antidiabetic, anti-inflammatory, and antioxidant properties. Studies show that rutin (50 mg/kg) reduces oxidative stress and plasma glucose levels by activating the nuclear factor erythroid 2-related factor 2 (Nrf2) signaling pathway, thereby alleviating diabetic neuropathy in rats [141]. The Nrf2 signaling pathway is essential for cellular defense mechanisms against inflammation and oxidative stress. Several cytoprotective and antioxidant genes are regulated to maintain cellular homeostasis [142]. In the same study, rutin was observed to protect organs commonly affected by diabetic complications. This protective effect is attributed to its ability to counteract diabetes-induced oxidative stress, preventing cardiac degeneration and improving electrocardiographic parameters compared to diabetic control rats [143]. Studies report that rutin demonstrates other biological activities, including antihypertensive, antithrombogenic, and antidepressant effects [94,144,145]. Oral administration of rutin reduces oxidative stress and inflammation in the heart by suppressing the NF-κB signaling pathway and upregulating *Nrf2* expression [146]. However, the potential toxicity of rutin remains a concern when considered for therapeutic use, as its safety profile has not been fully established. Studies show that these compounds induce oxidative stress [147] and cause mutagenesis in living systems [148]. Other studies report that rutin exhibits anti-mutagenic effects on cancer cells [149]. Given these contradictory findings, further rigorous research is required before rutin can be safely incorporated into medicinal or dietary formulations. This research is essential to ensure its safety and establish appropriate consumption or dosage levels.

## 3. Broad-Spectrum Pharmacological Activities of *G. Procumbens*

### 3.1. In Traditional Medicine

In China, *G. procumbens* has its unique name “Bai Bing Cao,” which means “100 ailments.” Studies involving animal models and in vitro studies have shown a range of properties which include cardioprotective, antihypertensive, antihyperglycemic, antioxidant, anti-inflammatory, anticancer, antimicrobial, organ-protective, and fertility-enhancing effects [14]. In Indonesia, this plant extract is widely used to manage renal dysfunction, while in Vietnam, it is employed in the treatment of fever. Additionally, in Thailand, inflammation and rheumatism are managed by this plant extract [14].

### 3.2. Antihypertensive Properties

In hypertensive rats, investigations have reported that *G. procumbens* significantly reduces systolic blood pressure and mean arterial pressure [150]. Additionally, *G. procumbens* extract significantly regulates other confounding factors of cardiovascular diseases, including heart rate, chronotropic, and ionotropic effects [14,150]. The renin-angiotensin system is crucial for vasoconstriction. The breakdown of angiotensin by renin is important for the regulation of blood pressure. *G. procumbens* extract exerts an inhibitory effect on angiotensin II activity [151]. However, there is a direct correlation between the blood pressure-lowering effect and the angiotensin-converting enzyme activity of *G. procumbens* [14]. Researchers have proposed that *G. procumbens* may antagonize angiotensin II, thereby inducing prostaglandin release and nitric oxide production [151]. Previous research has also observed that *G. procumbens* increases the level of nitric oxide in serum in hypersensitive rats [17]. Finally, owing to its involvement in several biological pathways, prominently the renin-angiotensin system, *G. procumbens* potentially serves to lessen hypertension.

### 3.3. Antiglycaemic Properties

As earlier mentioned, *G. procumbens* is widely used in traditional medicine for the treatment of diabetes mellitus. The hypoglycaemic effects of this plant extract have also been reported by researchers in animal model studies [152]. Furthermore, the observation that *G. procumbens* only induces a hypoglycaemic effect in diabetic rats but not in the control groups is quite intriguing [152]. To understand this observation, the effects of insulin secretion were further examined in vivo. However, no significant change in plasma insulin level was found in diabetic rats, which implies that the hypoglycaemic activity may be attributed to extra-pancreatic mechanisms rather than insulinotropic activity [153]. Previous studies reveal that *G. procumbens* extracts can stimulate the uptake of glucose in 3T3 adipocytes [154]. *G. procumbens* extracts may benefit individuals with glucose intolerance, while its ethanolic and aqueous extracts both reduce blood sugar levels, perhaps through AMP-activated protein kinase (AMPK)-mediated signaling pathways in muscle and adipose tissues [155]. The AMPK signaling pathway is a key metabolic regulator and a cellular energy sensor. It maintains cellular energy balance by promoting ATP-producing catabolic pathways and inhibiting ATP-consuming anabolic pathways [156]. In vivo studies show that *G. procumbens* regulates glucose uptake and utilization at peripheral levels in the muscle tissue of diabetic rats [157]. However, its molecular mechanisms in T2DM have remained unclear due to its complex composition [158]. Combination of *Azadirachta indica* A. Juss. and *G. procumbens* (Lour.) Merr. ethanolic extracts have produced a greater hypoglycemic effect than either herb alone. These two combined extracts could result in a potential blood glucose-lowering drug for individuals with diabetes [159]. The wide range of active compounds in the extract is responsible for the synergistic effect [159]. Collectively, the available data suggest the existence of bioactive components with insulin-mimicking qualities in *G. procumbens* [160].

### 3.4. Anticancer Properties

The frequent usage of *G. procumbens* in traditional medicine to treat cancers, including leukemia, uterine, and breast cancers, has stimulated the scientific community to dive deep into its antitumor properties [161]. It was discovered that a short-term (10-week) treatment utilizing the ethanolic extract of the plants inhibited the initiation phase of tongue carcinogenesis induced by nitroquinoline 1-oxide. High suppression of oral carcinogenesis was shown to result from a 26-week longer administration period [161]. *G. procumbens* was evaluated employing osteosarcoma cell lines. According to Wang et al., the treatment suppressed cancer cell invasiveness, migration abilities, and inhibited cell proliferation [162]. Another research showed that ethanolic extract of *G. procumbens* reduces azoxymethane-induced aberrant crypt foci by approximately 80%, thereby suggesting potential for the prevention of colon carcinoma [163]. Additionally, as evidenced in some studies, this plant extract has shown potential in effectively reducing tumors originating from mammary epithelial cells, thereby suggesting a positive role in preventing breast cancer [8,164]. *G. procumbens* inhibits the initiation phase of carcinogenesis mechanistically. Cytochrome P450 enzymes, including CYP3A4, show a marked decrease in expression and activity following treatment with the ethanolic extract [164]. This downregulation may contribute to a reduced risk of cancer occurrences [164]. Furthermore, it was revealed that *G. procumbens* therapy increases the expression of GST (glutathione-transferase), which is essential in detoxifying carcinogenic substances. Summarily, these mechanisms aid in chemoprevention [164].

Tumor cell growth, invasion, and metastasis are generally inhibited when angiogenesis pathways are blocked [165]. Studies reported that *G. procumbens* treatment inhibits the expression of vascular endothelial growth factor (VEGF) [166]. Furthermore, based on previous studies, *G. procumbens* can serve as a potential chemotherapeutic agent capable of targeting various cancer cell types [161]. Its effects are exerted by modulating different stages of the carcinogenesis process, including angiogenesis, metastasis, cancer initiation, and cell proliferation.

### 3.5. Antimicrobial Properties

Owing to the increasing prevalence of bacteria, viruses, and malaria strains that are resistant to currently available medications, alternative therapeutics are a prime necessity, with herbal therapeutics offering a promising solution. Extract of *G. procumbens* exhibits anti-plasmodial activity [167]. According to the study, *G. procumbens* extract exhibits chemosuppressive effects on malarial parasite strains (*Plasmodium falciparum* 3D7 and *Plasmodium berghei* NK65). Furthermore, studies have revealed that this plant extract exhibits virucidal and anti-replicative activities against HSV-1 and HSV-2, the herpes simplex viruses [168]. Clinical trials involving patients with herpes confirmed the effectiveness of *G. procumbens* extract-based herbal gels in managing the condition [168]. *G. procumbens* is also demonstrating bactericidal properties and could serve as a potential antibacterial agent against *Salmonella typhi*, *Pseudomonas aeruginosa*, *Vibrio parahaemolyticus*, and *Bacillus cereus* [14,169]. *G. procumbens* was also found to exhibit toxicity against fungi such as *Aspergillus niger* and *Candida albicans*. The findings of this study support the customary application of *G. procumbens* in pathogenic infection management, including malaria parasite strains (*Plasmodium falciparum* 3D7 and *Plasmodium berghei* NK65) and herpes simplex virus [14].

### 3.6. Antioxidant Properties

Antioxidant activity of *G. procumbens* extracts was assessed using the DPPH assay to quantify their capacity to scavenge free radicals [9]. Among the various types of plant extracts tested, the ethanol extract of *G. procumbens* exhibited the maximum potential in DPPH inhibition [170]. The ferric-reducing assay was also employed to quantify the reductive characteristic, further demonstrating the antioxidant capacity of the plant [7]. To distinguish the antioxidative capacity of different parts of *G. procumbens*, the root extract exhibited the highest level of antioxidant capacity among the other plant parts. Studies show that *G. procumbens* contains a high phenolic content, which may contribute to its powerful natural antioxidant activity [6].

### 3.7. Reproductive Function

Several studies have investigated the potential of *G. procumbens* in managing infertility. Sperm count and motility significantly increased in diabetic rats treated with *G. procumbens* extract, suggesting an association with the reproductive system [171]. *G. procumbens enhances* testicular lactate dehydrogenase activity, an enzyme critical for spermatogenesis [171]. Given the essential role of lactate dehydrogenase in spermatogenesis, this enhancement may contribute to improved fertility outcomes [172]. Overall, current evidence suggests that *G. procumbens* may support fertility in diabetic males, especially by increasing sperm count, quality, and motility.

### 3.8. Organ Protective Properties

Researchers have also examined whether *G. procumbens* can protect tissues and organs from damage [173]. Ethanolic extract reduces gastric ulcers in rats by decreasing submucosal edema and leucocyte infiltration, thereby providing a gastroprotective effect [174]. Researchers are now eager to further investigate the potential protective benefits of *G. procumbens*. A skin damage study on *G. procumbens* reported its antiphotoaging characteristics. It significantly inhibited the expression of matrix metalloproteinases in UV-irradiated human dermal fibroblasts [175]. Research findings reveal that the potential of *G. procumbens* to neutralize reactive oxygen species (ROS) may be related to its protective properties [6]. *G. Procumbens* is also widely recognized for its ability to inhibit the progression of renal illnesses. Aqueous extract of the plant was found to inhibit DNA synthesis and mesangial cell proliferation [61]. Furthermore, *G. procumbens* exhibited a hepatoprotective effect by reducing ethanol-induced lipid accumulation in the livers of mice through the modulation of genes related to lipid metabolism [176]. These results show that *G. procumbens* possesses considerable potential as an organ-protective agent. This effect is primarily attributed to its antioxidative qualities, which influence the regulation of gene expression [177]. In immunomodulation, *G. procumbens* functions either as a stimulant or suppressant, depending on the concentration administered in the experiment [178]. Consequently, inflammatory illnesses or immune system-related conditions may be treated with *the* anti-inflammatory and immunomodulatory properties of *G. procumbens*.

## 4. Modern Tools in Phytochemical Research

Over 25% of currently prescribed drugs are derived, either directly or indirectly, from plant sources, highlighting the enduring significance of medicinal plants in drug development [179]. Advancements in photochemistry, molecular biology, and computational tools have renewed interest in these natural reservoirs of structurally diverse and biologically active compounds [180]. Plant-derived natural products often serve as foundational molecules or pharmacophores in the design of novel therapeutics, particularly for complex diseases such as schizophrenia, neurodegenerative disorders, cancer, and metabolic diseases [181].

**Figure 3:**
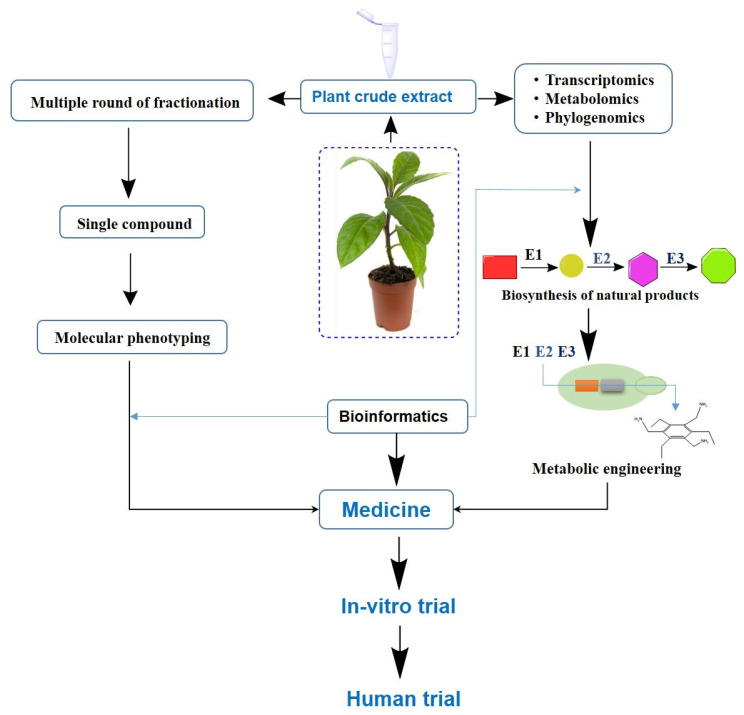
Schematic workflow of plant-derived drug discovery and development. The process begins with crude extracts derived from medicinal plants (e.g., *G. procumbens*), followed by multi-step fractionation, and molecular phenotyping. Concurrently, omics technologies (transcriptomics, metabolomics, and phylogenomics) elucidate biosynthetic pathways of natural products. Enzyme clusters (E1–E3) involved in metabolite biosynthesis are engineered through metabolic engineering to enhance compound production. Identified compounds are further developed into medicines, progressing through in-vitro trials and ultimately human clinical trials.

The integration of high-throughput screening (HTS), omics technologies, and in silico modeling has significantly accelerated the efficiency of discovering and refining bioactive compounds derived from plants (Figure 3) [182]. HTS enables the rapid assessment of thousands of phytochemicals for their potential activity against defined disease targets [183,184]. Advanced analytical techniques—including high-performance liquid chromatography (HPLC), gas chromatography–mass spectrometry (GC-MS), and liquid chromatography–mass spectrometry (LC-MS/MS)—allow for precise identification, quantification, and structural analysis of these compounds [185]. In silico tools—such as molecular docking, molecular dynamics simulations, and pharmacophore modeling—provide predictive insights into the binding affinities and interaction profiles of compounds with target proteins, thereby supporting lead optimization [186].

Omics technologies—including genomics, transcriptomics, metabolomics, and proteomics offer a comprehensive understanding of phytochemical biosynthesis and their underlying mechanisms of action [187]. Additionally, absorption, distribution, metabolism, excretion, and toxicity (ADMET) profiling, coupled with machine learning models, improves the identification of drug-like candidates with enhanced efficacy and safety profiles [188]. These interdisciplinary approaches have transformed modern drug discovery and significantly accelerated the development of plant-based medicines. However, further structural optimization is often required to improve pharmacokinetics, bioavailability, and target specificity. The successful translation of promising compounds into clinically effective agents, such as those derived from *G. procumbens*, depends on the seamless integration of computational drug discovery platforms. This process must be supported by rigorous in vitro and in vivo validation, comprehensive ADMET profiling, and systematic clinical evaluation [189].

## 5. Conclusions

*G. procumbens* demonstrates significant therapeutic potential and has long been regarded as a promising medicinal plant. Drawing on a comprehensive review of the current literature, this study highlights its broad pharmacological activities and reports minimal or negligible adverse effects, supporting its candidacy for alternative therapeutic development. However, further in-depth research is necessary to elucidate the interactions of *G. procumbens* extracts with key metabolic and signaling pathways. Such investigations could clarify the mechanisms underlying its therapeutic efficacy and enhance its pharmacological application. Ultimately, rigorous clinical trials that account for potential confounding variables are essential to confirm its safety and effectiveness in humans. Additionally, the formulation and standardization of *G. procumbens*-based therapeutic products remain significant challenges. However, the diverse phytochemical profile and proven bioactivities of the plant provide strong justification for its integration into the drug discovery pipeline. Therefore, coordinated, multidisciplinary research efforts are essential to fully realize the therapeutic potential of *G. procumbens*.

## Figures and Tables

**Table 2:** Bioactive compounds from *G. procumbens*.

Compound Class	Representative Compounds	Reported Activities	References
Flavonoids	Astragalin, Myricetin, Quercetin, kaempferol, and rutin	Antioxidant, antihypertensive, anti-inflammatory, and hepatoprotective activities	[17]
Phenolic acids	Caffeic acid, chlorogenic acid, ferulic acid, and gallic acid	Antioxidant, anti-diabetic, and antimicrobial activities	[25]
Saponins	Triterpenoid-type saponins	Immunomodulatory, hypolipidemic, and anticancer properties	[26]
Terpenoids and Sterols	β-Sitosterol, Stigmasterol, and Lupeol	Antidiabetic, anti-inflammatory, and cholesterol-lowering effects	[6]

**Table 3:** Regulation of several metabolic pathways by flavonoids and their therapeutic effects.

Flavonoids	Metabolic Pathway Regulated	Therapeutic Effect	References
Astragalin	Inhibits α-glucosidase and modulates the AMPK signaling pathway	Hyperglycemic activity	[28]
	Upregulates the expression and transcriptional activity of PGC1α, activates AMPK	Protective effect on mitochondrial quality control, and thus a promising drug candidate for the treatment of diabetic renal injury	[29]
	Inhibits the activity of CYP1B	Anti-tumor effect	[30]
	Inhibits HK2 through upregulating miR-125b	Inhibit the proliferation of hepatocellular carcinoma cells	[31]
	Downregulates the mRNA and protein expression of GLUT-1, LDH-A, and HK-2 in breast cancer cells	Anticancer potential in triple-negative breast cancer	[32]
	Inhibitory effect on PI3K/AKT signaling in gastric cancer	Anti-tumor effect	[33]
	Negatively modulates osteoclastogenesis via ROS and the MAPK signaling pathway	Preventive effect on inflammatory bone destruction	[34]
	Preserves blood-brain barrier integrity and inhibits neuroinflammation by modulating PK1/RIPK3/MLKL and mTOR/NF-κB pathways, thus alleviating LPS-induced depressive-like behaviors	Anti-inflammatory effect, potential to treat systemic inflammatory responses	[35]
Kaempferol	Upregulates anti-inflammatory cytokines	Anti-inflammatory effect	[36]
	Suppresses CD36 expression, mitochondrial membrane potential elevation, ROS production, MAPK/NF-κB expression, Ca^2+^ influx, and increases Nrf2/HO-1 levels in RAW264.7	Reduces atherosclerotic plaque formation	[37]
	Suppresses hepatocyte ferroptosis via Nrf2 pathway activation	Organ-protective effect	[38]
	Regulates the balance of Th17/Treg Cells and secretion of IL-17 and FoxO signalling pathways	Therapeutic effect against gouty arthritis	[39]
	Reduces the action of tumor necrosis factor-α (TNF-α) by returning the levels of LXR-α to their basal levels in human hepatocarcinoma cells	Anti-cancer effect	[36]
	Inhibitory effect on the activation of NF-κB and Akt in LPS plus ATP-stimulated cardiac fibroblasts decrease the release of TNF-α, IL-1β, IL-6, and IL-18.	Anti-inflammatory and cardio-protective effect	[40]
Myricetin	Inhibitory effect on matrix metalloproteinase 2 protein expression and enzyme activity	Anti-cancer effect	[41]
	Downregulates the mRNA expressions of T-bet and GATA-3 in Delphian lymph nodes	Immunomodulatory effect in atopic dermatitis	[42]
	Activates Sirt1 to regulate the JNK/Smad3 pathway	Ameliorate airway inflammation	[43]
	Induces apoptosis and autophagy in human gastric cancer cells through inhibition of the PI3K/Akt/mTOR pathway	Anti-cancer effect	[44]
	Induces apoptosis and autophagy by inhibiting PI3K/Akt/mTOR signaling	Anti-cancer effect in human colon cancer cells	[45]
Quercetin	Inhibits LPS-induced cytokine storm by interacting with the AKT1-FoxO1 and Keap1-Nrf2 signaling pathway in macrophages	Anti-inflammatory effect	[46]
	Attenuates high fructose feeding-induced atherosclerosis by suppressing inflammation and apoptosis via ROS-regulated PI3K/AKT signaling pathway	Reduce the atherosclerotic plaque	[47]
	Potently inhibits LPS-induced ROS and NO production in microglial cells	Anti-inflammatory effect	[48]
	Prevents THP-1 macrophage pyroptosis by reducing the expression of NLRP3 and cleaved-caspase1, as well as IL-1β and N-GSDMD in a concentration-dependent manner. Also, suppresses NLRP3 inflammasome activation by inhibiting ROS overproduction.	Anti-inflammatory effect	[49]
	Promotes the expression of LC3-II and beclin 1 and suppresses the expression of p62. Upregulate mRNA levels of LC3-II, Atg5, Atg7, and Atg12.	Anti-cancer effect	[50]
	Modulates NMDA-R mediated downstream signaling and PI3K/AKT-Nrf2/ARE signaling pathways in the hippocampus	Protect from cadmium-induced cognitive deficits in rats	[51]
Rutin	Downregulates p-AkT, p-ERK1/2, and p-mTOR	Promote growth arrest in A375 and C8161 melanoma cell lines	[52]
	Downregulates the NF-kB pathway and reduces pathological tau levels, regulates tau hyperphosphorylation by increasing PP2A levels	Potential therapeutic effect in Alzheimer’s disease	[53]
	Reduces gut microbiota dysregulation, such as the ratio of Firmicutes to Bacteroidetes, by regulating the AMPK/SREBP1 pathway	Ameliorate nonalcoholic fatty liver disease in diabetic patients	[54]
	Alleviates EndMT by restoring autophagy through inhibiting HDAC1 via PI3K/AKT/mTOR pathway in diabetic kidney disease	Delay the onset of diabetic kidney disease	[55]
	Inhibits the NLRP3 Inflammasome signaling pathway	Anti-inflammatory and anti-oxidant effects in ulcerative colitis	[56]

## Abbreviations

The following abbreviations are used in this manuscript:

SODE	superoxide dismutase
CAT	catalase
GPx	glutathione peroxidase
COX-2	cyclooxygenase-2
NF-κB	nuclear factor kappa-light chain enhancer of activated B cells
TNF-α	tumor necrosis factor-alpha
IL-6	interleukin-6
LDL	low-density lipoprotein
HDL	high-density lipoprotein
TLR4	Toll-like receptor 4
HO-1	heme oxygenase-1
MAPK	mitogen-activated protein kinase
BMP	bone morphogenetic protein
JAK/STAT	Janus kinase/signal transducer and activator of transcription
ATP	adenosine triphosphate
ROS	reactive oxygen species
VEGF	vascular endothelial growth factor
GST	glutathione S-transferase
AMPK	AMP-activated protein kinase
Nrf2	nuclear factor erythroid 2–related factor 2
HPLC	high-performance liquid chromatography
GC-MS	gas chromatography–mass spectrometry
LC-MS/MS	liquid chromatography–mass spectrometry tandem mass spectrometry
HTS	high-throughput screening
ADMET	absorption, distribution, metabolism, excretion, and toxicity
